# Determination of Urinary Biomarkers for Assessment of Short-Term Human Exposure to Aflatoxins in São Paulo, Brazil

**DOI:** 10.3390/toxins6071996

**Published:** 2014-07-08

**Authors:** Alessandra V. Jager, Fernando G. Tonin, Pollyana C. M. C. Souto, Rafaela T. Privatti, Carlos A. F. Oliveira

**Affiliations:** 1 Department of Food Engineering, School of Animal Science and Food Engineering, University of São Paulo, Pirassununga, SP 13635-900, Brazil; E-Mails: alejager@usp.br (A.V.J.); pollyanasouto@usp.br (P.C.M.C.S.); rafaela.privattiu@usp.br (R.T.P.); 2 Department of Biosystems Engineering, School of Animal Science and Food Engineering, University of São Paulo, Pirassununga, SP 13635-900, Brazil; E-Mail: fgtonin@usp.br

**Keywords:** AFM_1_, AFB_1_, exposure assessment, mass spectrometry

## Abstract

In the present study, a longitudinal assessment was carried out to evaluate the short-term human exposure to aflatoxins in Pirassununga region, São Paulo, Brazil, by determination of urinary aflatoxins by a liquid chromatography coupled to mass sprectrometry (UPLC-MS/MS) method. Sixteen volunteers with ages ranging from 14 to 55 years old were instructed to collect the early morning first urine four times every three months, from June 2011 to March 2012, totaling 64 samples. Aflatoxin M_1_ (AFM_1_) was found in 39 samples (61%) at levels ranging from 0.19 to 12.7 pg·mg^−1^ creatinine (mean: 1.2 ± 2.0 pg·mg^−1^ creatinine). Residues of aflatoxins B_1_, B_2_, G_1_, G_2_ and aflatoxicol were not identified in any urine sample. No significant difference was found among the AFM_1_ mean levels in urine samples collected in the four sampling periods. The levels of AFM_1_ found in urine samples indicate a low short-term exposure of the population studied to aflatoxins through the diet, although further investigations are needed to assess other long-term biomarkers of exposure to AFB_1_.

## 1. Introduction

Aflatoxins are secondary metabolites produced by fungi of the genus *Aspergillus*, mainly by species *A*. *flavus*, *A*. *parasiticus* and *A*. *nomius*. These fungi naturally grow in food products and are able to initiate a variety of toxic effects in vertebrates, including humans [1]. The prevalence of tropical climate in Brazil creates ideal conditions for the development of these fungi, mainly in cereal products [2]. The main types of aflatoxins that can be found in plant substrates are identified as B_1_, B_2_, G_1_ and G_2_. Aflatoxin B_1_ is the most toxic compound, and its primary biotransformation in the liver undergoes detoxification reactions, which generate aflatoxins M_1_, Q_1_, B_2a_, P_1_ and aflatoxicol (AFL) [3]. These metabolites and their parent, non-metabolized compounds can ultimately be excreted in urine and feces [4,5]. Aflatoxin M_1_ (AFM_1_) is also found in milk of lactating cows that have consumed feeds contaminated with AFB_1_. In 1987, the International Agency for Research on Cancer classified AFB_1_ in Group 1-human carcinogen [6]. In past decades, several studies have demonstrated that residues of aflatoxins and their metabolites in human urine are valuable biomarkers of short-term aflatoxin exposure through the diet. Correlations between ingestion of AFB_1_ and excretion of AFM_1_ in urine were observed in Guangxi province, People’s Republic of China, Egypt and in Gambia [7,8,9]. 

The occurrence of aflatoxins in Brazilian foodstuffs has been frequently reported, mainly in peanut and corn grains at rates from up to 50% of the samples analyzed [10,11]. However, there is little information on the human exposure to aflatoxins as assessed by aflatoxin biomarkers in human urine in Brazil, except for two reports, one of them by Romero *et al*. [12], who observed AFM_1_ in 65% out of 65 urine samples from inhabitants of the city of Piracicaba, state of São Paulo with values ranging from 1.8 to 39.9 pg·mL^−1^ and mean concentration of 5.96 pg·mL^−1^. In another recent study, Giolo *et al*. [13] detected AFM_1_ in 37.2% of 43 urine samples from chronic carriers of hepatitis B virus (HBV), and 17.2% of 29 samples from a HBV non-carriers control group. Determination of residual aflatoxins in urine in these previous works has been carried out using high performance liquid chromatography (HPLC) with fluorescence detection, and at a single sampling time. The objective of the present study was to assess the human exposure to aflatoxins in Pirassununga region, state of São Paulo, Brazil, through the determination of AFM_1_, AFL, and residues of AFB_1_, AFB_2_, AFG_1_ and AFG_2_ in urine using a validated method by LC coupled to mass spectrometry (MS/MS), at four sampling times over a 10-month period.

## 2. Results and Discussion

### 2.1. Method Performance

**Figure 1:**
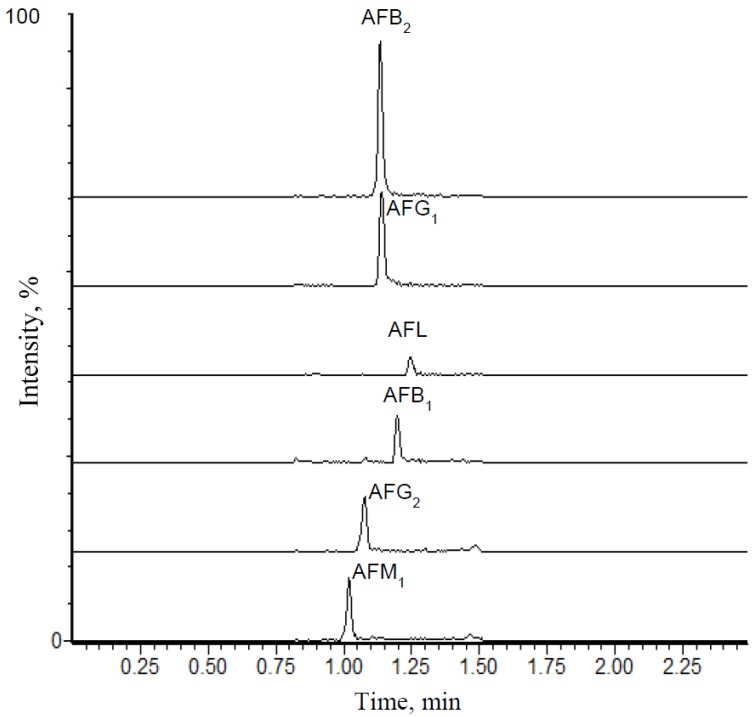
Chromatogram (quantification transitions) of a standard mixture containing 5 pg·mL^−1^ of aflatoxins B_1_, B_2_, G_1_, G_2_, M_1_, and aflatoxicol.

Table 1 shows the recovery and precision results estimated for all aflatoxins analyzed in urine samples. LOD and LOQ values for AFB_2_ and AFG_1_ were 0.03 and 0.10 pg·mL^−1^, respectively. For AFB_1_, AFM_1_, AFG_2_ or AFL, the LOD and LOQ values were 0.075 and 0.25 pg·mL^−1^, respectively. Calibration curves of all the aflatoxins showed coefficients of determination higher than 0.99, and residuals plot did not show tendency or deviation from linearity. Recoveries values were higher than 67% for all analytes and coefficient of variation ranged from 2% to 28%. Figure 1 shows a chromatogram of a standard mixture containing 5.0 pg·mL^−1^ of each aflatoxin. Taking into account that different quantification and confirmatory transitions were used for AFB_2_ and AFG_1_, there were no interferences related to co-migration of these two aflatoxins in any chromatographic run.

**Table 1:** Performance characteristics of the analytical method for determination of aflatoxins B_1_, B_2_, G_1_, G_2_, M_1_ and aflatoxicol in urine samples.

Analyte	LOD (pg·mL^−1^)	LOQ (pg·mL^−1^)	Spiking Level ^a^ (pg·mL^−1^)	Recovery ^a^ (%)	CV ^b^ (%)
AFM_1_	0.075	0.25	0.25	87	27
			2.50	80	6
			5.00	79	4
AFB_1_	0.075	0.25	0.25	83	25
			2.50	78	14
			5.00	78	10
AFB_2_	0.03	0.10	0.10	95	20
			2.50	81	11
			5.00	95	4
AFL	0.075	0.25	0.25	88	22
			2.50	87	2
			5.00	84	3
AFG_1_	0.03	0.10	0.10	73	28
			2.50	81	3
			5.00	78	13
AFG_2_	0.075	0.25	0.25	75	15
			2.50	67	7
			5.00	70	4

LOD: Limit of detection; LOQ: Limit of quantification; ^a^ Mean recovery, *n* = 6; ^b^ Coefficient of variation, *n* = 6.

The analytical method developed was successfully validated, providing satisfactory results for the determination of residues of AFB_1_, AFB_2_, AFG_1_ and AFG_2_ and the metabolites AFM_1_ and aflatoxicol in urine. Special attention was given to reduce the limit of detection without decreasing recovery and precision, making feasible the first investigation of residues of AFB_1_, AFB_2_, AFG_1_, AFG_2_ and aflatoxicol in urine from Brazilian population. Furthermore, in Brazil this is the first assessment of AFM_1_ in urine by LC-MS/MS, where unequivocal identification of target analytes is provided. 

One precursor ion and two fragment ions were used for the confirmation of each analyte, resulting in four identification points. This is higher than the minimum requirement of three identification points for the confirmation of mycotoxins, as stated by Directive 96/23/EC [14]. 

### 2.2. Analysis of Urine Samples from Volunteers

**Figure 2:**
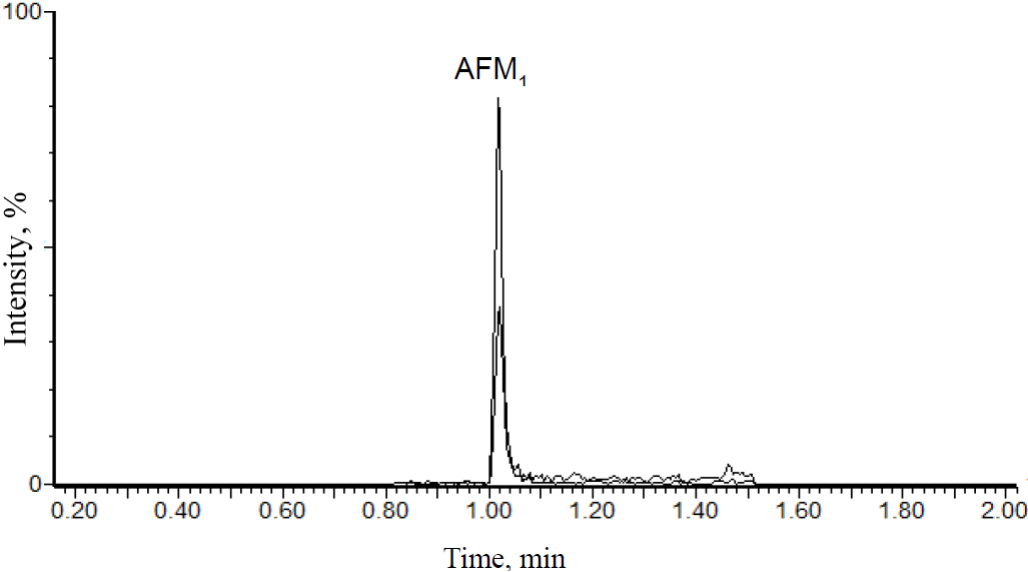
Chromatogram of a urine sample naturally contaminated with 0.44 pg·mL^−1^. Quantification transition (329 > 273) is represented by the major signal and confirmatory transition (329 > 243) by the minor signal, respectively.

Results of AFM_1_ determined for each volunteer are presented in Table 2, along with mean values, standard deviation and variations of AFM_1_ concentration for positive samples in each sampling event, calculated and expressed as pg·mg^−1^ creatinine. Figure 2 shows a chromatogram of a positive urine sample for AFM_1_. None sample had quantifiable concentrations of AFB_1_, AFB_2_, AFG_1_, AFG_2_ and AFL. Residues of AFB_1_, AFB_2_, AFG_1_, and AFG_2_ are not commonly found in human urine samples, however, quantifiable concentrations were found in individuals from some regions of China, Egypt and Guinea with high exposure to dietary aflatoxins [8,15]. Data reported in China did not mention the mean or range of AFB_1_ concentrations found in urine samples [15]. However, the mean levels of urinary aflatoxins found in Egyptian children (*n* = 50) were 189, 1.4, 76.6 and 2.2 pg·mL^−1^, for AFB_1_, AFB_2_, AFG_1_ and AFG_2_, respectively [8]. In another survey conducted in Guinea, children (*n* = 50) also shown AFB_1_ levels in urine up to 18,000 pg·mL^−1^, with mean concentrations of 2.6, 5.7, 26.6 and 19.0 pg·mL^−1^ for AFB_1_, AFB_2_, AFG_1_ and AFG_2_, respectively [8]. The absence of AFL residues in urine in the present study is consistent with a previous report describing undetectable levels of AFL in urine samples from children in Egypt [16]. Moreover, AFL residues have been found in feces from children with Kwarshiokor [17] in human serum [16] and human placenta [18]. 

AFM_1_ was found in 39 samples (61%) at levels ranging from 0.19 to 12.7 pg·mg^−1^ creatinine (mean: 1.2 ± 2.0 pg·mg^−1^ creatinine). The mean AFM_1_ level found in human urine samples from Pirassununga region is much lower than the concentrations reported in early assessments conducted in areas with high incidence of aflatoxins in foods, especially in China [7,15]. Tang *et al*. [19] conducted an intervention study with green tea in China, and found AFM_1_ levels ranging from 0.42 to 141.9 pg·mg^−1^ creatinine at the beginning of the study. Mykkanen *et al*. [5] found AFM_1_ concentrations in urine ranging from 10 to 33 pg·mL^−1^ in China. In a recent evaluation accomplished in Malaysia, AFM_1_ mean concentration of 23.4 ± 17.7 pg·mL^−1^ was reported by Redzwan *et al*. [20]. These values are much lower than former studies on the incidence of AFM_1_ in human urine in those countries, usually up to 5200 pg·mL^−1^ [7,15], but still higher than those observed in Brazil in the present study. 

**Table 2:** Aflatoxin M_1_ levels in urine samples collected from volunteers ^1^.

Individual	Sampling A June 2011 (*N* = 16)		Sampling B September 2011 (*N* = 16)		Sampling C December 2011 (*N* = 16)		Sampling D March 2012 (*N* = 16)	
	pg·mL^−1^	pg·mg^−1^ Creatinine	pg·mL^−1^	pg·mg^−1^ Creatinine	pg·mL^−1^	pg·mg^−1^ Creatinine	pg·mL^−1^	pg·mg^−1^ Creatinine
Females								
1	3.9	2.0	0.70	0.67	4.2	2.0	0.46	0.27
2	3.5	2.3	ND	ND	1.20	1.2	0.36	0.28
3	3.1	2.2	ND	ND	1.20	1.1	0.36	0.19
4	ND	ND	1.7	2.2	1.30	1.9	ND	ND
5	0.38	0.31	ND	ND	ND	ND	ND	ND
6	0.48	1.4	1.2	2.3	0.50	0.68	ND	ND
7	0.75	1.6	ND	ND	0.65	0.49	0.25	0.54
8	ND	ND	0.27	0.57	ND	ND	0.27	0.41
Mean ± SD	2.0 ± 1.6	1.6 ± 0.7	1.0 ± 0.6	1.5 ± 1.0	1.5 ± 1.4	1.2 ± 0.6 ^a^	0.3 ± 0.1	0.3 ± 0.1
Median	1.9	1.8	1.0	1.5	1.2	1.2	0.4	0.3
Range	0.38–3.9	0.31–2.3	0.27–1.7	0.57–2.3	0.50–4.2	0.49–2.0	0.25–0.46	0.27–0.54
*n* (%) ^2^	6 (75)		4 (50)		6 (75)		5 (63)	
Males								
9	0.7	0.25	1.1	0.44	1.0	0.44	0.35	0.21
10	ND	ND	6.9	12.7	ND	ND	ND	ND
11	0.3	0.40	ND	ND	ND	ND	ND	ND
12	ND	ND	ND	ND	0.44	0.34	ND	ND
13	0.8	0.58	0.53	0.38	0.56	0.35	ND	ND
14	0.78	2.24	ND	ND	ND	ND	ND	ND
15	0.52	0.38	0.91	0.50	ND	ND	0.29	0.19
16	5.2	2.8	0.60	0.24	0.76	0.20	1.3	0.66
Mean ± SD	1.5 ± 1.9	1.3 ± 1.1	2.2 ± 2.7	3.0 ± 5.0	0.6 ± 0.2	0.3 ± 0.1 ^b^	0.8 ± 0.6	0.4 ± 0.3
Median	0.74	0.49	0.91	0.44	0.66	0.35	0.35	0.21
Range	0.52–5.2	0.25–2.8	0.53–6.9	0.24–12.7	0.44–1.0	0.20–0.44	0.29–1.3	0.19–0.66
*n* (%) ^2^	7 (88)		5 (63)		4 (50)		3 (38)	
Total								
Mean ± SD	1.7 ± 1.7	1.4 ± 0.9	1.5 ± 2.0	2.0 ± 4.0	1.2 ± 1.1	1.0 ± 1.0 ^a^	0.5 ± 0.4	0.3 ± 0.2
Median	0.77	1.5	0.91	0.57	0.89	0.59	0.36	0.27
Range	0.38–5.2	0.25–2.8	0.27–6.9	0.24–12.7	0.44–4.2	0.20–2.0	0.25–1.3	0.19–0.66
n (%) ^2^	12 (75)		9 (56)		10 (63)		8 (50)	

^a,b^ In the same column, means followed by different letters present significant difference (*p* < 0.05); ^1^ Results expressed in pg·mg^−1^ creatinine; ^2^ Number of positive samples; ND: Not detected (Limit of quantification: 0.25 pg·mL^−1^).

The mean concentration of AFM_1_ found in urine samples in the present assessment (1.3 ± 1.5 pg·mL^−1^) was lower than previous assessments carried out in Brazil. Romero *et al*. [12] analyzed 69 urine samples and observed that 65% of them were positive for AFM_1_, with a mean value of 5.96 ± 6.39 pg·mL^−1^. Giolo *et al*. [13] found the AFM_1_ mean concentration for positive samples of 2.74 ± 1.98 pg·mL^−1^ (*N* = 72; range for positive samples: 0.67–7.87 pg·mL^−1^). These previous works reported data from single sampling times, while in the present study samplings were carried out four times every three months during a 10-month period. No difference (*p* > 0.05) was found between mean AFM_1_ levels in urine collected in the four sampling events. Although the mean AFM_1_ levels in urine samples from males in 3rd sampling (C) were higher than females (*p* = 0.0227), no important variations regarding to the gender in the human exposure to aflatoxins were observed in the present study.

Experimental evidence from studies with animals indicates that the excretion of AFM_1_ in urine is proportional to the amount of AFB_1_ ingested in the contaminated diet [21,22]. The correlation between AFB_1_ ingested and AFM_1_ excreted urine has also been demonstrated for humans. An epidemiological study conducted in China found AFM_1_ concentration in the range of 0 to 3200 pg·mL^−1^ in urine from volunteers with a daily intake in the range of 4 to 221 µg·day^−1^ [7]. Zhu *et al*. [7] also estimated that AFM_1_ excreted in urine was in the range of 1.23%–2.18% of total AFB_1_ ingested for male population, and 1.30%–1.78% for the female population, respectively. Taking into account the mean values of carry-over rates as estimated by Zhu *et al*. [7] (1.7% for males and 1.5% for females), and the mean AFM_1_ levels excreted in urine by volunteers in the present study (1.28 and 1.27 pg·mL^−1^ for males and females, respectively), Probably Daily Intake (PDI) values were estimated considering the mean volume of urine collected from volunteers (330 mL for both males and females) and the mean body weight (75 kg and 64 kg for males and females, respectively). The resulting PDI for males and females were 0.34 ng·kg^−1^ body weight (b.w.) day^−1^ and 0.42 ng·kg^−1^ b.w. day^−1^, respectively. These PDI values are lower than the PDI value of 1.58 ± 2.5 ng·kg^−1^ b.w. day^−1^ estimated by Jager *et al*. [23], based on Food Frequency Questionnaires and the analysis of food products in the same region of the present study. The comparison between PDI values obtained from different methods is difficult, especially because in the present study one early morning urine sample was collected, hence representing a limited period of urine volume excreted by volunteers. However, when using the highest AFM_1_ value found in urine for males (6.9 pg·mL^−1^) and females (4.2 pg·mL^−1^) groups, the worst cases of aflatoxin ingestion would be 1.8 ng·kg^−1^ b.w. day^−1^ and 1.4 ng·kg^−1^ b.w. day^−1^, respectively, which are similar to the mean PDI value as described by Jager *et al*. [23]. 

Although the carry-over of AFB_1_ in foods to AFM_1_ in urine may vary according to the level of toxin ingested and physiological differences, among other factors [21,24], the low levels of AFM_1_ found in the present study indicate a low human exposure to aflatoxins in the population studied. Therefore the low levels of aflatoxins in the diet of volunteers may be a consequence of annual variation in the aflatoxin occurrence in foodstuffs, or better quality control practices in the manufacture of food products in the state of São Paulo, to comply with the regulations for aflatoxins established in 2011 [25]. In Brazil, good manufacturing practices (GMP) have become compulsory for peanut industries since 2003, when the National Health Surveillance Agency issued the regulation [26]. Accordingly, low levels of up to 36.7 µg·kg^−1^ of total aflatoxins (B_1_ + B_2_ + G_1_ + G_2_) in peanut and corn based products and 0.069 µg·L^−1^ of AFM_1_ in liquid milk consumed in the same region studied has been reported before [23,27]. 

However, the fact that AFB_1_ and AFM_1_ are potent hepatocarcinogens warrants concern about the human exposure levels through the diet, as assessed by other long-term biomarkers of exposure to AFB_1_ such as serum AFB_1_-lysine adduct in the population studied.

## 3. Experimental Section

### 3.1. Sampling Design

The study was conducted in households at Pirassununga region, state of São Paulo, Brazil from June 2011 to March 2012. Residents (*N* = 16; 8 males and 8 females) with ages ranging from 14 to 55 years old were invited to participate as volunteers, providing urine samples four times every three months, totaling 64 samples. Volunteers were instructed to collect the early morning first urine in a polyethylene vessel previously supplied. Samples were maintained in a cool box until transport to the laboratory, where they were separated in 50 mL aliquots and kept frozen at −20 °C until analysis. The total volume of urine was measured and the body weight of each individual was recorded. The experiment was approved by the ethics committee of the School of Medicine at Ribeirão Preto, University of São Paulo. 

### 3.2. Chemicals and Materials

All reagents were of analytical grade and water was purified by deionization (Milli-Q system, Millipore, Bedford, MA, USA). HPLC-grade acetonitrile and methanol (JT Baker, Xalostoc, Mexico) were used for chromatographic analyses. The aflatoxin standards AFB_1_, AFB_2_, AFG_1_, AFG_2_, AFM_1_ and AFL were purchased from Sigma (Sigma, St. Louis, MO, USA). Individual stock solutions were prepared in acetonitrile, evaluated according to Scott [28] (AOAC method 971.22), and stored in an amber glass vial at −20 °C. Individual aflatoxins B_1_, B_2_, G_1_, G_2_, M_1_ and AFL solutions were mixed in convenient volumes of methanol:water (50:50, *v/v*) to produce working solutions at concentrations starting from limit of quantitation determined for each aflatoxin and 25, 50, 75 and 100 pg·mL^−1^, of each aflatoxin. 

### 3.3. Determination of Aflatoxins in Urine Samples by UPLC-MS/MS

Samples were thawed at room temperature and centrifuged at 3500 rpm for 10 min. Twenty milliliter of sample was diluted with 20 mL of sodium acetate buffer (pH 5.0), and the mixture eluted through an immunoaffinity column Aflatest, (Vicam, Watertown, MA, USA) which was washed with 40 mL of distilled water and the retained aflatoxins eluted with 1 mL of methanol. Extract was evaporated at 40 °C until dryness and dissolved in 1 mL methanol:water (50:50, *v/v*). Extracts were analyzed on a Waters Acquity I-Class UPLC system (Waters, Milford, MA, USA) equipped with a BEH C_18_ column (2.1 × 50 mm, 1.7 μm) and coupled to a Xevo TQ-S mass spectrometer (Waters, Milford, MA, USA). The column was kept at 40 °C during analyses, and samples were maintained at 10 °C. Ten microliters of samples extracts and standards were injected. Gradient elution was employed with mobile phase composed by water (eluent A) and acetonitrile (eluent B), both containing 0.05% of aqueous ammonia. After an initial period of 0.2 min at 90% A, the percentage of B was linearly raised to 60% over 1.1 min (1.3 min). Then, eluent B was increased to 90% over 0.05 min, followed by a hold time of 0.25 min (1.60 min). Following this, the percentage of B was reduced to 10% over 0.1 min (1.7 min) and the column re-equilibrated to initial conditions for 0.8 min. Total chromatographic run time was 2.5 min and the mobile phase flow rate was maintained at 0.6 mL·min^−1^. The mass spectrometer was operated in Multi Reaction Monitoring (MRM) mode using electrospray ionization in positive ion mode, with a capillary voltage of 0.75 kV, a source temperature of 150 °C and a desolvation temperature of 650 °C. Desolvation gas flow and cone gas flow were maintained at 500 L·h^−1^ and 150 L·h^−1^, respectively. Cone voltage, collision energy and MRM transitions (major precursor ion > fragment ion) were automatically optimized for each compound individually using Intellistart program (Waters, Milford, MA, USA) of Acquity UPLC console. The most intense transition was set for quantification and the second most intense was used as confirmatory transition. Quantification MRM transitions were *m*/*z* 313 > 285, 315 > 287, 329 > 243, 331 > 245, 329 > 273, 297 > 269 for AFB_1_, AFB_2_, AFG_1_, AFG_2_, AFM_1_ and AFL, respectively. Confirmatory transitions were *m/z* 313 > 241, 315 > 259, 329 > 200, 331 > 189, 329 > 229, 297 > 115 for AFB_1_, AFB_2_, AFG_1_, AFG_2_, AFM_1_ and AFL. Table 3 contains the mass spectrometer conditions of analytes. Data collection and processing was performed using software MassLynx version 4.1 (Waters, Milford, MA, USA). 

**Table 3:** Mass spectrometer conditions for detection of analytes by Multi Reaction Monitoring (MRM) method.

Analyte	Precursor Ion (*m*/*z*)	Product Ions (*m*/*z*)	Cone Voltage (V)	Collision Energy (V)
AFB_1_	313	285 ^a^	94	36
		241 ^b^	94	22
AFB_2_	315	287 ^a^	2	26
		259 ^b^	2	28
AFG_1_	329	243 ^a^	2	26
		200 ^b^	2	38
AFG_2_	331	245 ^a^	56	28
		189 ^b^	56	40
AFM_1_	329	273 ^a^	52	24
		229 ^b^	52	38
AFL	297	269 ^a^	40	20
		115 ^b^	40	58

Notes: ^a^ Transitions used for quantification; ^b^ Transitions used for confirmation; V, voltage.

### 3.4. Method Validation for Determination of Aflatoxins Residues in Urine

Calibration curves of the aflatoxins were prepared at concentration ranges of 5.0 to 100 pg mL^−1^ for AFB_1_, AFG_2_, AFM_1_ and AFL, and 2.0 to 100 pg mL^−1^ for AFB_2_ and AFG_1_. These calibration levels were prepared considering the 20-fold pre-concentration of samples during the extraction procedures, hence corresponding to concentrations in urine samples of 0.25 to 5.0 pg·mL^−1^ for AFM_1_, AFB_1_, AFL and AFG_2_, and 0.1 to 5.0 pg·mL^−1^ for AFB_2_ and AFG_1_. The limits of detection (LOD) and quantification (LOQ) were calculated for each method of analysis based on signal:noise ratio of 3:1 and 10:1, respectively. Linearity was evaluated by verifying the coefficient of determination (*r*^2^) and visual inspection of residuals plot. Triplicate injection of each concentration level was used. Recovery and precision were assessed by adding a mixture of standards to blank urine to generate fortified samples with concentrations of 0.25, 2.5 and 5.0 pg·mL^−1^ of each aflatoxin. All fortified samples were prepared and extracted in six replicates and final concentration was calculated by triplicate injection of each extract. 

### 3.5. Determination of Creatinine in Urine Samples

Urinary creatinine levels were determined by a kinetic alkaline picrate (Jaffe reaction) method, using a commercial Test Kit (Labtest, Lagoa Santa, Brazil). Photometric measurements were performed using Spectrumlab 22 PC (Leng Quang Tech., Shanghai, China) at 510 nm. 

### 3.6. Statistical Analysis

Results obtained in each sampling period were submitted to variance analysis by using the SAS^®^ proc mixed (SAS, 2004, Cary, NC, USA), in order to verify differences among AFM_1_ concentrations in urine at different sampling times. The statistically significant difference was calculated with a probability level of 0.05 (*p* < 0.05).

## 4. Conclusions

Results of this trial indicate a high incidence of AFM_1_ at low levels in urine samples in Pirassununga region, state of São Paulo, Brazil, while AFB_1_, AFB_2_, AFG_1_, AFG_2_, and AFL were not detected in any sample. The concentrations of AFM_1_ found in urine samples of volunteers indicate a low short-term exposure of the population studied to aflatoxins through the diet over a 10-month period. However, further investigations are needed to assess other long-term biomarkers of exposure to AFB_1_ in the population under study.
